# Immortalization and Targeted Enrichment of HIV-Infected CD4^+^ T-Cells from Patients Under Antiretroviral Therapy

**DOI:** 10.3390/ijms27021086

**Published:** 2026-01-22

**Authors:** Whitney E. Bruchey, Sharada Paudel, Ashley L. McCormack, Tomozumi Imamichi, Sylvain Laverdure

**Affiliations:** 1 Laboratory of Human Retrovirology and Immunoinformatics, Frederick National Laboratory, Frederick, MD 21702, USA; whitney.bruchey@nih.gov (W.E.B.); ashley.mccormack@nih.gov (A.L.M.); timamichi@mail.nih.gov (T.I.); 2 Biopharmaceutical Developmental Program, Frederick National Laboratory, Frederick, MD 21702, USA; sharada.paudel@nih.gov

**Keywords:** HIV-1, CD4^+^ T-cells, cloning, patient-derived, immortalization, defective proviruses

## Abstract

Defective HIV-1 proviruses harboring mutations and/or large internal deletions represent the majority of HIV-1 sequences found in circulating peripheral blood mononuclear cells of people living with HIV with viremia suppressed by combination antiretroviral therapy; indirect evidence suggests that such sequences are transcriptionally active and may contribute to immune activation. In this study, we present a new approach allowing for high-efficiency screening, immortalization, and targeted enrichment of HIV-positive CD4^+^ T-cells isolated from people living with HIV. Using this method, we were able to isolate and expand patient-derived cells, identify mutations and deletions via sequencing, and confirm that those proviruses were transcriptionally and translationally active in vitro. Moreover, our findings indicate that the majority of proviral sequences circulating in suppressed HIV-infected patients may undergo 3′-LTR deletions, suggesting that sequence diversity reported using LTR-to-LTR amplification and sequencing approaches may indeed be underscored.

## 1. Introduction

Despite the long-term suppression of the plasma viral load in the majority of people living with HIV (PLWH) on combination antiretroviral therapy (cART), HIV-1 provirus-bearing cells continue to persist, creating a significant obstacle in HIV-1 eradication. Those persisting proviral sequences have been divided into replication-competent and defective proviruses: while both types can be found in patients with measurable viral loads, defective sequences comprise most proviruses isolated from suppressed (i.e., <40 copies per milliliter) PLWH [1,2]. Although defective proviruses were initially believed to be of little clinical significance, considering their inability to fit in the canonical HIV replication cycle, recent studies have highlighted their potential relevance in comorbidities associated with long-term HIV infection. In fact, while active proviral transcription during suppressive ART has been previously described [3], defective HIV-1 proviruses specifically have been shown to not only be transcriptionally active in vivo [1,4] but also to produce viral proteins [5], while in PLWH this protein expression has been shown to trigger a specific immune response [6]. Considering that chronic immune activation persists in ART-suppressed PLWH, preventing CD4^+^ T-cell count restoration [7], and that defective HIV-1 sequences account for over 90% of proviruses isolated from these patients, this could therefore suggest that defective proviruses might be at least partially responsible for the persistent inflammation seen in the setting of treated chronic HIV-1 infection [8].

The expression profile and functional analysis of viral proteins and potential novel chimeric proteins translated from defective proviruses may thus provide further insight into the persistence of the immune activation observed in suppressed patients. However, while defective proviruses have the coding potential to express canonical and non-canonical viral proteins, in vivo expression and functional assessment of these viral proteins are yet to be determined, mainly due to the lack of a culture system that supports the long-term survival of patient-derived CD4^+^ T cells. In this study, we used Herpesvirus saimiri (HVS), a T cell tropic γ-Herpesvirus of nonhuman primates, to immortalize CD4^+^ T-cells isolated from patients undergoing cART. Earlier publications have demonstrated that HVS-immortalized T cells could be maintained in culture for extended period of times without further antigen or mitogen stimulation [9], while retaining normal function phenotype [10] and antigen specificity [11]. Such an approach has been used previously to immortalize and clone CD4^+^ cells from HIV-infected individuals; however, most of the recovered clones were HIV negative [12]. Therefore, we developed a new approach combining high-yield cell sorting, early screening for HIV positivity, and overall improved culture conditions, allowing for the high-efficiency generation of immortalized HIV-infected CD4^+^ T cell isolated from PLWH with suppressed viremia.

## 2. Results

### 2.1. High-Throughput Screening and Sequencing of HIV-1-Positive Wells

While earlier reports have reported the immortalization of peripheral blood lymphocytes from bulk HVS infection, resulting in immortalized CD4^+^ and CD8^+^ T-cell recovery [13], these methods were not deemed suitable for our purpose, for two major reasons: first, this approach preferentially selected CD8^+^ cells from PLWH individuals, likely due to their serological profile [12,13], and more importantly very of the immortalized clones were actually HIV-infected. Considering our specific focus on HIV-infected patient-derived cells, we needed to improve this method by (i) adding an early screening for HIV positivity to phase out immortalized uninfected wells and (ii) automating the plating process to increase the throughput and hence the likelihood of HIV-positive cell recovery.

**Figure 1:**
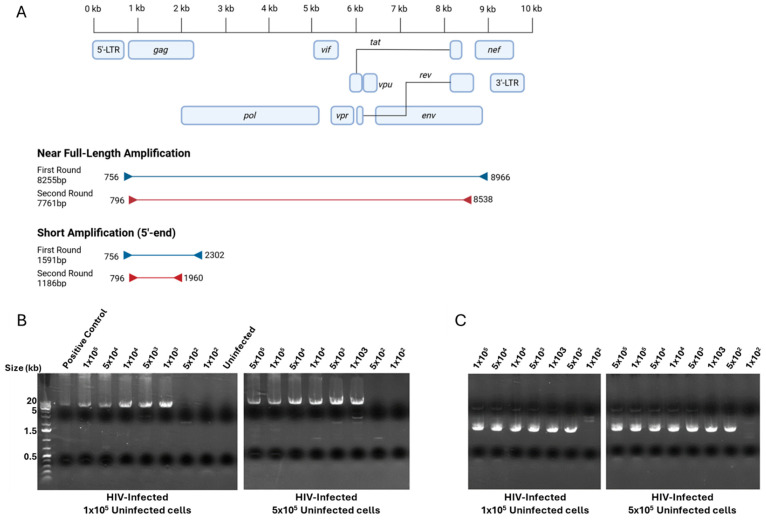
PCR amplification strategy and detection limits. (**A**) Representation of primers and amplicon sizes for first- and second-round short and near full-length amplification; primer coordinates are based on HXB2 reference sequence. Created in BioRender. Bruchey, W. (2026) https://BioRender.com/fr4repk (accessed on 12 December 2025). (**B**) Agarose gel images presenting detection limits of nested PCR amplification of near full-length primer sets. Ratios of NL4-3.Luc.E- infected cells ranging from 1 × 10^2^ to 1 × 10^5^ cells spiked in 1 × 10^5^ and 5 × 10^5^ uninfected CD4^+^ T cells. (**C**) Representation of primers selected for first- and second-round short amplification. Ratios of NL4-3.Luc.E- infected cells ranging from 1 × 10^2^ to 1 × 10^5^ cells spiked in 1 × 10^5^ and 5 × 10^5^ uninfected CD4^+^ T cells.

In order to allow for the high-throughput screening of HIV-positive wells, we elected to use a high-fidelity DNA polymerase, usable on crude cell lysates, thereby waiving the need for DNA isolation and purification. First, we tested several combinations of primer sets near the 5′- and 3′-LTR from published [5] and custom-designed primers, scoring each set based on the length, amplification, sensitivity, and reduction in non-specific binding (Table 1, Table 2 and Table 3, Supplementary Figure S1). To increase the sensitivity, we decided that a nested PCR approach was needed. We choose forward primer 756 and reverse primer 8966 for first-round near full-length PCR (Figure 1A). Following that nested PCR strategy, we again tested combinations of primer sets internal from the chosen first-round forward and reverse primers (Table 2). We chose forward primer 796 and reverse primer 8538 for second-round near full-length PCR (Figure 1A). Although defective proviral sequences have been shown to bear mutations and/or large internal deletions, HIV-1 *gag* is notably conserved [2], prompting our decision to establish a short-length primer set specific to the *gag* region. Again, we chose forward primers 756 and 796 for first- and second-round PCR and tested reverse primers at the *gag* 3′-end (Table 3, Figure 1B, Supplementary Figures S2 and S3). We chose reverse primers 2303 and 1960 for short-length first- and second-round PCR (Figure 1B). These highest scoring primer sets were evaluated for their detection limits by assessing the amplification of HIV-infected cells at multiple ratios: 1 × 10^2^ to 1 × 10^5^ in vitro NL4-3.Luc.E- infected cells were mixed with 1 × 10^5^ or 5 × 10^5^ uninfected cells, and both near full-length and short amplifications were carried out (Figure 1B,C, Supplementary Figures S4 and S5). We determined that our approach would allow for the detection of 1000 and 500 infected cells for the long and short amplification strategies, respectively. All positive wells were subsequently subjected to LTR-to-LTR amplification and sequencing following a previously published method [1]. However, most proviruses could not be amplified using this approach, warranting the development of an alternate sequencing method. Cells detected with the short-length primer sets yielded an amplicon of approximately 1200 bp within *gag* and were not suitable for the prediction of potential viral protein expression. In preparation of sequencing HIV-1 positive cells obtained using the short-length primer set, we established a series of 24 reverse primers spanning from the 5′-LTR to the 3′-LTR, as previously published [5] (Supplementary Figures S6 and S7); in this instance, the longest amplicon obtained from PCR amplification was used as sequencing template. On the other hand, for wells screened positively using the near full-length primer set, amplicons were used directly as sequencing template. All HIV-1-positive templates were sequenced using a series of 48 reverse and forward primers spanning the HIV-1 proviral genome within LTR regions [5]. The resulting sequences from HIV-1 positive cells were aligned to the HXB2 reference and subjected to open reading frame (ORF) analysis.

### 2.2. Immortalization and Targeted Enrichment of Patient-Derived HIV-Infected CD4^+^ T-Cells

**Figure 2:**
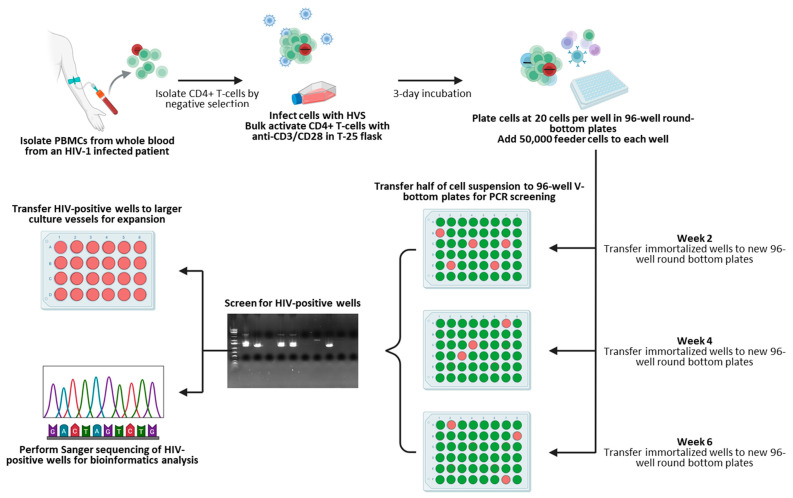
Isolation, sorting, and screening workflow. Created in BioRender. Bruchey, W. (2026) https://BioRender.com/ckkfl5c (accessed on 16 December 2025).

We next focused on combining our screening approach with a high-throughput immortalization and sorting method using CD4^+^ T cells purified from two HIV-infected individuals, summarized in Figure 2. Both donors had undetectable viral loads at the time of collection, as shown in Table 4. PBMCs were isolated from whole blood draws using gradient centrifugation, subsequently followed by the purification of CD4^+^ T-cells, as previously described [14]. CD4^+^ T-cells were then infected with HVS at a multiplicity of infection (MOI) of 0.5 and activated in a flask coated with anti-CD3/anti-CD28 antibodies for three days. One day prior to sorting, PBMCs from heterologous healthy blood donors were irradiated and seeded into 96-well round-bottom plates at 50,000 cells per well as feeder cells, providing activation and promoting cell growth. Activated and HVS-infected CD4^+^ T-cells were then harvested and plated at 20 cells per well in 96-well round-bottom culture plates using a Namocell Pala single-cell dispenser. A total of 18 and 84 plates were prepared for patients 1 and 2, respectively, according to the number of cells recovered at the time of sorting. CD4^+^ T-cells were subsequently kept in culture with a combination of IL-2 and antiretroviral drugs (NNRTI: Efavirenz, protease inhibitor: Darunavir, and integrase inhibitor: Dolutegravir) to prevent reinfection events and maintain proviral clonality; the cell growth was monitored, and wells displaying cell growth were harvested for HIV screening as described in the Methods section. A summary of those numbers, including HIV positivity, is displayed in Table 5, and a complete breakdown of HIV-positive well screening results is shown as Supplementary Table S1. Although the cell viability over time was not assessed in this instance, prior experiments demonstrated that in vitro HIV-infected cells could survive and sustain growth for more than 105 days following HVS infection (Supplementary Figure S8), confirming previous findings describing HVS infections as an effective way to immortalize T-cells from PLWH. Interestingly, only a small number of recovered provirus-bearing cells showed positivity using the near full-length screening method (2 out of 19 for patient 1 and 8 out 35 for patient 2), suggesting that many circulating HIV-infected CD4^+^ T-cells might potentially have undergone 3′-LTR mutations and/or deletions in those two patients.

**Figure 3:**
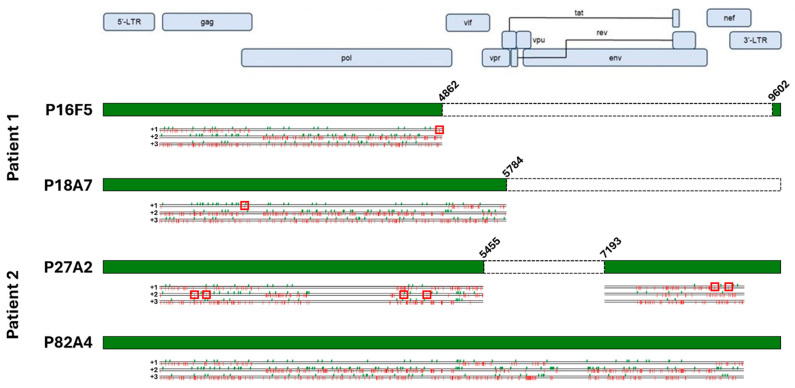
Sequence analysis of immortalized patient-derived cells. Sequencing results for each isolated proviral sequences (green boxes) are aligned to the HXB2 reference sequence, along with terminal coordinates. Internal deletions are indicated by white boxes. Start (green checkmarks) and stop (red bars) codons in all three frames are indicated below each sequence. Premature stop codons with HIV genes are highlighted with red squares. Created in BioRender. Bruchey, W. (2026) https://BioRender.com/8h27lbz (accessed on 12 December 2025).

We chose to focus the downstream experiments on two HIV-positive wells from each patient donor: for patient 1, we randomly selected well P16F5, positive for both screening methods, and well P18A7, only showing positivity for the short primer set, while for patient 2, two double-positive wells were chosen, namely P27A2 and P82A4. Proviruses were sequenced as described in the Methods section. Since we lacked LTR-to-LTR amplification for P18A7, we used an inverse PCR approach, as described previously [2,5], to determine the proviral integration site and used flanking genomic primers to amplify the provirus, after which sequencing was carried out normally. Using this approach, we determined that the P18A7 proviral sequence was integrated in chromosome 15, specifically in the intronic region spacing the first two exons of the *smad3* gene. The sequencing results are displayed in Figure 3. We found that both patient 1-derived infected cells had 3′-LTR deletions. Indeed, P18A7 lacked the entirety of the 3′-end of the proviral genome, with the proviral sequence abruptly stopping at position 5782. ORF analysis of P18A7 revealed that this clone also presented a premature stop codon within the *gag* gene, potentially impacting Gag expression and likely abolishing GagPol translation; *vif* and *vpr* genes lacked mutations, while all downstream regulatory, accessory, and envelope genes had been deleted. Conversely, the proviral sequence for P16F5 was even shorter, with a large internal deletion spanning nucleotides 4863-9601, suppressing all viral regulatory and accessory genes. Additionally, while the *gag* sequence for P16F5 lacked non-sense mutations, a premature stop codon was present in *pol*, likely suppressing GagPol expression. Similarly, for patient 2, we found that P27A2 also had a large internal deletion spanning nucleotides 5456-7193, suppressing *vif*, *vpr*, *vpu*, *tat*, *rev,* and *nef* genes; the remaining proviral sequence was also hypermutated, with several premature stop codons in *gag*, *pol*, and *nef*, likely entirely abolishing any viral gene expression in those cells. In contrast, we found that P82A4 was full-length and lacked gene-suppressing mutations and/or deletions; this was a surprising finding considering that both patients had undetectable viral loads at the time of blood collection.

### 2.3. HIV-1 Proviruses in Immortalized Cells Are Transcriptionally and Translationally Active

**Figure 4:**
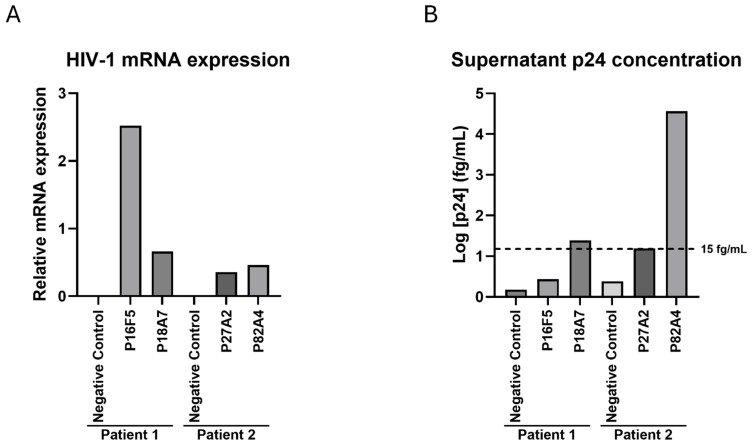
HIV-1 mRNA and p24 levels in immortalized HIV-infected cells. (**A**) Total RNA was extracted from each well, and total HIV-1 mRNA and GAPDH levels were measured; results show relative HIV-1 mRNA normalized by GAPDH expression for each well, and results from one representative experiment are shown. (**B**) Cells were kept in culture for four weeks, half of culture medium was changed every week, then supernatants were harvested and assessed for HIV-1 p24 concentration. The experimental assay cutoff of 15 fg/mL is represented on the graph. Results from one representative experiment are shown.

We next sought to establish whether LTR transcriptional activity was retained in our in vitro culture conditions for those four HIV-1-positive cells, despite the observed mutations and deletions. Total RNA was extracted, and the total HIV-1 mRNA levels were measured as described in the Methods section, while the GADPH levels were used as internal controls; HIV-1-negative immortalized CD4^+^ T-cells from each patient were used as negative controls (Figure 4A). We determined that all four proviruses displayed transcriptional activity in immortalized cells, while no measurable viral mRNA could be detected from either negative control. Notably, of those four infected cells, P16F5 showed the highest level of transcriptional activity. Further investigation into LTR activation, including into potential mutations affecting transcription factor response elements as well as presence and functionality of regulatory proteins would be needed to explain these differences. Furthermore, we wanted to determine whether viral particles could still be found in the culture supernatants, despite the mutations previously described. Therefore, the cells were kept in culture for four weeks in the presence of IL-2 and antiretroviral drugs, after which the supernatants were collected, and the p24 concentrations were assessed using the Simoa technology as described in the Methods section. We found that of the four assessed wells, only two displayed a measurable level of capsid protein in their culture supernatants. The P18A7 p24 levels were just above the assay positivity cutoff at 24.3 fg/mL, while larger quantities of p24 were present in the P82A4 supernatant (36,296.8 fg/mL); neither the P16F5 nor P27A2 p24 levels rose above the 15 fg/mL detection limit (Figure 4B).

## 3. Discussion

Our study adapted previously published approaches to immortalizing and isolating HIV-infected CD4^+^ T-cells from infected individuals: we devised a two-way screening method allowing for the detection of both full-length and hypermutated proviral sequences with high sensitivity, and we also applied and automated a previously published T-cell immortalization method [13] allowing for the culture of low-frequency cells such as HIV-infected CD4^+^ T-cells. Half of the proviruses characterized in this study presented partial or complete 3′-LTR deletions, a type of in vivo mutation which has been rarely reported [15]. Moreover, out of the 54 HIV-positive wells we obtained from these two patients, less than 20% (10 out of 54) could be directly amplified from LTR to LTR, underscoring the frequency of 3′-LTR mutations and/or deletions in those patients. Further analysis is needed to determine exactly which type of proviral modification is present in each HIV-infected cell. We were able to demonstrate that the immortalized cells nonetheless could retain transcriptional and translational activities, which does suggest they could be a viable tool to study defective provirus-bearing cells’ protein expression profiles ex vivo; however, this approach should not be construed as a surrogate for in vivo viral transcription analysis, since LTR activity might be impacted by cellular immortalization. Interestingly, it is worth noting that out of the three defective proviral sequences we isolated, only P18A7 displayed measurable p24 levels in the cell culture supernatant. It is likely that the lack of a functional *env* genes in all the defective sequence-bearing cells impacted their ability to release viral particles, which would explain our findings. Additional functional analysis on a larger number of cells bearing defective provirus is certainly warranted to assess their ability to produce and release virions after immortalization.

Although this method was initially developed for the long-term culture of patient-derived cells, focusing on in vitro analysis of viral protein expression and potential pro-inflammatory phenotypes, we found that the enrichment and culture of these cells allowed us to find a new type of underreported sequence diversity. Indeed, among the immortalized HIV-positive cells we further characterized, two had 3′-LTR deletions, either complete or partial. Most current HIV sequencing methods use either a full LTR-to-LTR amplification strategy [1,5,8,16,17], a droplet digital PCR (ddPCR) approach [18,19], or a combination of both methods [20] to sequence proviral genomes or assess genomic integrity. While ddPCR would allow for the detection of 3′-LTR deletions and would be the preferential tool to assess prevalence of those deletions in vivo, in would not be suitable for further downstream testing such as complete proviral sequencing or integration site analysis. Conversely, approaches solely using LTR-to-LTR amplification will limit their analysis and sequence diversity estimates to those proviral sequences retaining their primer target sequences.

While also PCR-based and hence not fully unbiased, our screening results suggest that the majority of immortalized HIV-positive cells we obtained either entirely lacked or were bearing 3′-LTR mutations, which would have prevented their identification solely using a conventional LTR-to-LTR amplification. Using an additional shorter PCR screening approach allowed us to identity those cells as positive, while our immortalization and targeting enrichment method provided the extended culture time needed for further sequencing and characterization. We believe this approach should be expanded to further characterize proviral sequence diversity in PLWH: we suggest that future experiments based on our immortalization and enrichment method should use multiple short amplicons spanning the entirety of the provirus for the detection of HIV-positive cells, rather than a single-PCR approach limiting the detection of positive wells to those proviruses containing intact target sequences. Combined with sequencing of the entirety of the cellular genome to avoid PCR-based sequencing limitations due to hypermutated or deleted sequences, this could allow for a fully unbiased analysis of the diversity of proviral sequences in patients undergoing antiretroviral therapy.

Interestingly, LTR deletions have been largely documented for other retroviruses such as HTLV-1: in this case, while both 5′- and 3′-LTRs can be deleted in patients suffering from adult T-cell leukemia [21], deletion of the 5′-LTR specifically has been linked to oncogenesis [22]. However, very few mentions of HIV-1 3′-LTR deletions in vivo have been reported in the literature, although evidence of LTR asymmetry in patients undergoing antiretroviral therapy has been published previously [23]. Additionally, several reports have originated from a cohort of patients infected with a strain of HIV-1 containing deletions within the *nef* gene and the 3′-LTR [24,25]. Considering that survivors of these infections remained asymptomatic for over 14 years without any antiretroviral therapy, these deletions and mutations have been associated with reduced pathogenicity [26]. It is, therefore, worth noting that over 80% (44 out of 54) of the sequences isolated from patients with undetectable viral loads through our approach had similar 3′-LTR deficiencies, as demonstrated by their lack of LTR-to-LTR amplification permissiveness. Our data strongly suggest that further analysis is critical to determine how prevalent those mutations are in patients with different immunological and serological status; our screening method would be of interest for those future projects.

## 4. Materials and Methods

### 4.1. CD4^+^ T Cell Isolation and HVS Infection and Activation

Peripheral blood mononuclear cells (PBMCs) were isolated from HIV-infected patients’ whole blood by gradient centrifugation using lymphocyte separation medium (MP Biomedicals), and the CD4^+^ T cells were subsequently isolated from PBMCs using the EasySep human CD4^+^ T cell isolation kit (Stemcell Technologies, Vancouver, BC, Canada). One day before isolation, one well in a 6-well plate was coated with 2 mL of 10 μg/mL Mouse Anti-Human CD3 antibody and 10 μg/mL Mouse Anti-Human CD28 antibody (BD Biosciences, San Jose, CA, USA) in 1X PBS (Quality Biological, Gaithersburg, MD, USA) overnight at 37 °C; the coated well was washed three times with 1X PBS before cell plating. Isolated CD4^+^ T cells were transferred to the well at 2 × 10^6^ cells per mL of RP-AIMV10 culture medium, which is comprised of a 1:1 ratio of RPMI 1640 medium (Thermo Fisher Scientific, Waltham, MA, USA) and AIM-V medium (Thermo Fisher Scientific) supplemented with 10% heat-inactivated Fetal Bovine Serum (FBS) (R&D Systems, Bio-techne, Minneapolis, MN, USA), 10 mM 4-(2-hydroxyethyl)-1-piperazineethanesulfonic acid (HEPES), pH 7.4 (Quality Biological, Gaithersburg, MD, USA), and 5 µg/mL of Gentamycin (Thermo Fisher Scientific). Cells were then infected with HVS at a multiplicity of infection of 0.5 and cultured for three days at 37 °C.

### 4.2. Herpesvirus Saimiri Culture and Isolation

HVS strain C-488 (ATCC VR-1414) was cultured in Owl Monkey Kidney (OMK) cells (ATCC CRL-1556) for generation of an HVS C488 viral stock. OMK cells were initially seeded into a T-25 flask at 5 × 10^4^ cells/cm^2^ in 5 mL of Dulbecco’s Modified Eagle’s Medium (DMEM, Thermo Fisher Scientific) supplemented with 10% heat-inactivated Fetal Bovine Serum (FBS, R&D Systems, Bio-techne), 10 mM 4-(2-hydroxyethyl)-1-piperazineethanesulfonic acid (HEPES), pH 7.4 (Quality Biological), and 5 µg/mL of Gentamycin (Thermo Fisher Scientific), hereafter referred to as D10 medium. Once confluent, cells were harvested using 0.25% trypsin/0.1% EDTA solution (Quality Biological) for five minutes at 37 °C, split between two T-75 flasks, and cultured until confluent, with media changes occurring every three to four days. Two days prior to infection, confluent OMK cells were cultured at a 1:3 ratio in 15 mL of complete medium in a T-75 flask. Cells were infected with HVS strain C488 (ATCC VR-1414) using 10 infectious particles per flask, cultured for four hours at 37 °C; then, an additional 10 mL of culture medium was added to the flask, and the cells were cultured for 7 to 14 days. The virus-containing supernatant was collected when most cells had detached from the bottom of the T-75 flask and were centrifuged at room temperature for 5 min at 300× *g* for removal of cellular debris. The supernatant was aliquoted into 1.5 mL microcentrifuge tubes and stored at −80 °C until use.

### 4.3. Titration of HVS Viral Stock

OMK cells were seeded in a 96-well flat-bottom tissue culture plate in 100 μL of the above-described culture medium at 10,000 to 15,000 cells per well. A 1 mL aliquot of HVS viral stock was removed from −80 °C, thawed at room temperature and subjected to serial 10-fold dilutions in complete medium, ranging from 10^−1^ to 10^−8^. Then, 100 μL of each dilution was added per well to each row of the OMK cell-seeded plates; the plates were sealed with parafilm and incubated for two weeks at 37 °C. Titration of the virus was then achieved by observation of the cytopathic effect (CPE): the plates were observed for CPE, and the viral titer was determined based on the fraction of CPE-positive wells across all dilutions using the Spearman–Kärber approximation, where titer = 10^(Sum of fractions+0.5)^ [27].

### 4.4. Preparation of NL4-3.Luc.E- Virions

VSV-G-pseudotyped HIV-luciferase virions (NL4-3.Luc-E- were prepared by co-transfection of HEK293T cells with pNL4-3ΔEnv-Luc [28,29] and pLTR-VSVG [30] using the TransIT-293 transfection reagent (Mirus, Pyrmont, New South Wales), as previously described [30]. Cell culture supernatants were harvested 48 h after transfection and filtered with 0.45 μm Steriflip Filter Units (MiliporeSigma, Darmstadt, Germany). Virus particles were pelleted by ultracentrifugation at 100,000× *g* for 2 h, at 4 °C on a 20% Sucrose in 150 mM NaCl-HEPES, pH 7.4 buffer, and resuspended in antibiotics-free D10 medium. The virus concentration was quantified by a p24 antigen capture kit (PerkinElmer, Waltham, MA, USA), and aliquots were stored at −80 °C until use.

### 4.5. Cell Sorting and Targeted Enrichment of HIV-Positive Cells

Twenty-four hours before cell sorting, PBMCs from a heterologous healthy donor were irradiated at 3000 rad (30 Gy) and plated as feeder cells at 5 × 10^4^ cells per well in round-bottom 96-well plates. Activated HVS-infected cells were recovered after 3 days of culture, diluted in RP-AIMV10 medium to 1 × 10^5^ cells/mL, and supplemented with 2.5 nM Calcein AM (Namocell) as a viability dye. After a 10 min room-temperature incubation, cells were further diluted to 5 × 10^4^ cells/mL; then, 20 cells per well were dispensed using the Namocell Pala automated cell sorter (Bio-Techne). Cell viability was assessed prior to sorting using an FSC/TSC and Calcein AM gating strategy, according to the manufacturer’s instructions. Following sorting, cells were maintained in RP-AIMV10 culture medium supplemented with 50 U/mL IL-2 (R&D Systems), 10 nM dolutegravir, 10 nM darunavir, and 10 nM efavirenz (Selleckchem, Houston, TX, USA) to prevent viral re-infection and maintain proviral clonality. The cell growth was monitored for a minimum of 6 weeks; about half the cells of wells displaying growth were recovered biweekly and used for HIV PCR screening as described below. HIV-positive wells were further expanded in larger culture vessels for downstream applications.

### 4.6. PCR Screening of HIV-1 Proviruses

To screen wells for HIV-1 positive cells, cells were transferred to 96-well V-bottom plates, lysed with 120 μL DirectPCR lysis reagent and proteinase K (Viagen Biotech, Los Angeles, CA, USA), and placed on a shaker at 56 °C for 5 h. Cell lysates were then transferred to 96-well PCR plates and placed on a thermocycler at 85 °C for 45 min. PCR amplification was performed using the VeriFi high fidelity DNA polymerase (PCR Biosystems, London, England), according to the manufacturer’s instructions. Near full-length nested PCR amplification was carried out as follows: for the first PCR round, we used an initial denaturation at 95 °C for 2 min, followed by 35 cycles of denaturation at 95 °C for 10 s, annealing at 64 °C for 15 s, and extension at 72 °C for 5 min, with the final extension at 72 °C for 10 min, using primers FWD: 756 5′-CGGCGACTGGTGAGTACGCCAA-3′ and REV: 8966 5′-CCTCCTCCTCTTGTGCTTCTAGCC-3′. The second near full-length amplification run used primers FWD: 796 5′-GCGAGAGCGTCAGTATTAAGC-3′ and REV: 8538 5′-CAATCAAGAGTAAGTCTCT-3′, under the above-described conditions. The short-length PCR amplification primers were performed similarly, with a shortened extension step at 72 °C for 1 min, using PCR primers FWD: 756 5′-CGGCGACTGGTGAGTACGCCAA-3′, 5′ and REV: 2302 CTAATAGAGCTTCCTTTAGTTGCC-3′ for the first round amplification and primers FWD: 796 5′-GCGAGAGCGTCAGTATTAAGC-3′ and REV: 1960 5′-CTTTGCCACAATTGAAACACTT-3′ for the second round. The lysate of PCR detected HIV-positive cells was further amplified using previously published [1] LTR-to-LTR amplification primers FWD: F1 5′-AAATCTCTAGCAGTGGCGCCCGAACAG-3′ and REV: R1 5′-TGAGGGATCTCTAGTTACCAGAGTC-3′ for first round amplification and primers FWD: F3 5′-ACAGGGACCTGAAAGCGAAAG-3′ and REV: R3 5′-CTAGTTACCAGAGTCACACAACAGACG-3′ for the second round at a lengthened extension step at 72 °C for 5 min. PCR reactions were performed in volumes of 25 μL in 0.2 mL 96-well PCR plates; the final primer concentrations in all reactions were 400 nM. PCR products were purified using QIAamp purification kits (Qiagen, Hilden, Germany) prior to sequencing.

### 4.7. Inverse PCR

This approach was adapted from a previously published method [2,5], allowing for identification of both genomic sequences flanking the provirus. Genomic DNA (2 μg) from well P18A7 was digested using 20U BclI-HF (New England Biolabs, Ipswich, MA, USA) for 16 h at 37 °C. Following heat activation for 20 min at 65 °C, 200 ng of digested DNA was ligated using 0.4 U T4 DNA ligase (New England Biolabs) for 16 h at 16 °C. The ligated DNA was then heat inactivated at 65 °C for 15 min and placed on ice. The primers used to detect the 5′ integration site were FWD: +2165 5′-CAGAAGAGAGCTTCAGGTTTGGG-3′ and REV: -576 5′-GATCTCTAGTTACCAGAGTCA-3′ for first round amplification and FWD: +2208 5′-TCAGAAGCAGGAGCCGATAGAC-3′ and REV: −558 5′-GTCACACAACAGACGGGCACAC-3′ for second round amplification. The primers used to detect the 3′ integration site were FWD: +4162 5′-CACACAAAGGAATTGGAGGAAATG-3′ and REV: −2691 5′-TATGGATTTTCAGGCCCAATTTTTGA -3 for first round amplification and FWD: +4745 5′-TAAGACAGCAGTACAAATGGCAG-3 and REV: −2603 5′-GGCCATTGTTTAACTTTTGGG-3′ for second round amplification. Each PCR round was performed using the VeriFi high fidelity DNA polymerase (PCR Biosystems), with the initial denaturation at 95 °C for 2 min, followed by 35 cycles of denaturation at 95 °C for 10 s, annealing at 60 °C for 15 s, and extension at 72 °C for 1 min 30 s, with the final extension at 72 °C for 10 min.

### 4.8. HIV-1 Sequencing

PCR products of near full-length amplicons or the longest amplicons obtained from the series of 24 reverse primers were sequenced with PCR primers spanning the HIV-1 genome (Table 6, Supplementary Figure S1) using the 3500xL Genetic Analyzer (Applied Biosystems, Waltham, MA, USA) and the BigDye Terminator v3.1 Cycle Sequencing Kit (Thermo Fisher Scientific), according to the manufacturer’s instructions. Sequencing reads were aligned to the HXB2 reference sequence (GenBank K03455) using Sequencher (Gene Codes Corporation, Ann Arbor, MI, USA) and analyzed for mutations.

### 4.9. Viral mRNA Quantification

Quantitative analysis of the HIV-1 mRNA levels was performed as previously described [31]. Total RNA from CD4^+^ T-cells was isolated after 65 days of culture using the RNeasy Isolation kit (Qiagen). First, 1 mg of total RNA was used to generate cDNA using random hexamers, and Taqman Reverse Transcription Reagents (Thermo Fisher Scientific); samples were run on a QuantStudio 7 Pro system. The total transcribed HIV mRNA levels were quantified using a primer and probe set against the U5 region of the virus (FWD: 5′-TGTGTGCCCGTCTGTTGTGT-3′, REV: 5′-GAGTCCTGCGTCGAGAGAGC-3′, PROBE: 5′-(FAM)-CAGTGGCGCCCGAACAGGGA-(TAMRA)-3′) and normalized by glyceraldehyde 3-phosphate dehydrogenase (GAPDH) mRNA levels (Hs99999905_m1, Applied Biosystems).

### 4.10. Supernatant p24 Quantification

Levels of the HIV-1 p24 protein in culture supernatants were determined on a Simoa^®^ HD-X analyzer using the Simoa^®^ HIV p24 Advantage Kit (Quanterix, Billerica, MA, USA). The cutoff for this assay on culture supernatants, calculated as 2.5 standard deviations from the mean signal obtained from HIV-negative supernatants, has been determined to be 15 fg/mL, which is lower than previously established on plasma samples [32]; nine reference calibrators and two supplied quality controls (low and high concentrations) were included in each run. Samples were processed undiluted following the manufacturer’s instructions. Standard curves for each run were created by analyzer software to calculate four-parameter curve fit, 1/y2 weighted, and then used for logistic regression fitting to quantify the concentration of p24 samples. Sample results above the assay dynamic range, set by the cutoff and calibration curves, were diluted in 3% BSA (Sigma-Aldrich, St. Louis, MO, USA) in 1X PBS (Quality Biological) and run again.

## Figures and Tables

**Table 1:** PCR primers tested for detection of HIV-1 positive cells.

Primer Location	Sequence (5′-3′)
F1	AAATCTCTAGCAGTGGCGCCCGAACAG
F2	TCTCTCGACGCAGGACTCGGCTTG
F3	ACAGGGACCTGAAAGCGAAAG
+711	CGCAGGACTCGGCTTGCTGAAG
+756	CGGCGACTGGTGAGTACGCCAA
+796	GCGAGAGCGTCAGTATTAAGC
+836	GGGAAAAAATTCGGTTAAGGCC
+1127	AAAAGGCACAGCAAGCAGCAGCT
−1631	TTTGGTCCTTGTCTTATGTCCAGAATGC
+1546	AATCCACCTATCCCAGTAGGAGAAAT
−1960	CTTTGCCACAATTGAAACACTT
+2078	AGGCTAATTTTTTAGGGA
+2165	CAGAAGAGAGCTTCAGGTTTGGG
−2138	TGTTGGCTCTGGTCTGCTCT
−2302	CTAATAGAGCTTCCTTTAGTTGCC
−2603	GGCCATTGTTTAACTTTTGGG
+2603	CCCAAAAGTTAAACAATGGCC
−2691	TATGGATTTTCAGGCCCAATTTTTGA
−2872	TGCATCACCCACATCCAGTA
−3246	CCATTTATCAGGATGGAGTTC
−3299	TGTCATTGACAGTCCAGCTG
+3194	CACACCAGACAAAAAACATCAG
+3379	RGCAATTATGTAAACTCCTTAGGGGA
−3501	TAAGTCTTTTGATGGGTCATAATA
+3869	CTATGTAGATGGGGCAGCTA
−4176	TCTACTTGTTCATTTCCTCC
+4024	AAGTAAACATAGTAACAGACTCAC
+4162	CACACAAAGGAATTGGAGGAAATG
+4745	TAAGACAGCAGTACAAATGGCAG
−4956	TACTGCCCCTTCACCTTTCCA
−5195	TAGTGGGATGTGTACTTCTGAAC
+5550	AGAGGATAGATGGAACAAGCCCCAG
−5562	TGGTCTTCTGGGGCTTGTTC
−5833	AGGGCTCTAGTCTAGGATCTACTGGCTCCA
−5857	GGCTGACTTCCTGGATGCTTCCAGGGC
+5861	TGGAAGCATCCAGGAAGTCAGCCT
−6207	CTCTCATTGCCACTGTCTTCTGCTC
+6210	CAGAAGACAGTGGCAATGA
−6214	CACTCTCATTGCCACTGTCT
−6342	TTCCACACAGGTACCCCATA
+6445	GTGTACCCACAGACCCCAACCCACAAG
−6952	TTCCATGTGTACATTGTACTGTG
+6952	GCACAGTACAATGTACACATGGAA
−7515	GGCATACATTGCTTTTCC
+7515	GGAAAAGCAATGTATGCC
−7795	ATAGTGCTTCCTGCTGCTC
+7626	TTCAGACCTGGAGGAGGAGATATG
+7795	GAGCAGCAGGAAGCACTAT
+7850	ACAATTATTGTCTGGTATAGTGCAACAGCA
−8538	CAATCAAGAGTAAGTCTCT
+8277	TTCATAATGATAGTAGGAG
−8868	TCCCACCCTATCTGCTGCTGGC
−8882	TCTCGAGATGCTGCTCCCACCC
−8966	CCTCCTCCTCTTGTGCTTCTAGCC
−9039	GGCTAAGATCTACAGCTGCCTTG
−9081	GGAGTGAATTAGCCCTTCCAGTCCC
−9146	CTGCCAATCAGGGAAGTAGCCTTGTGT
−9604/R2	GCACTCAAGGCAAGCTTTATTGAGGCTTA
R3	CTAGTTACCAGAGTCACACAACAGACG
R1	TGAGGGATCTCTAGTTACCAGAGTC

Coordinates based on HXB2 reference sequence. The symbol “+” or “F” indicates forward orientation, with reverse orientation as “−“ or “R”.

**Table 2:** First- and second-round primer sets for near full-length HIV-1 amplification.

**First-Round Primer Sets**					
**Forward**	**Reverse**	**Amplicon Size (bp)**	**Amplification**	**Single Band**	**Specificity**
F1	R1	9064	✓	x	x
F1	R3	9054	✓	x	x
F1	9604	9010	✓	x	x
F1	9146	8549	✓	x	x
F1	8538	7934	✓	x	x
F1	7795	7191	✓	x	x
F1	7515	6910	✓	x	x
F2	R1	9005	✓	x	x
F2	R3	8995	✓	x	x
F2	9604	8951	✓	x	x
F2	9146	8490	✓	x	x
F2	8538	7875	✓	x	x
F2	7795	7132	✓	x	x
F2	7515	6851	✓	x	x
F3	R1	9041	✓	x	x
F3	R3	9031	✓	x	x
F3	9604	8987	✓	x	x
F3	9147	8526	✓	x	x
F3	8538	7911	✓	x	x
F3	7795	7168	✓	x	x
F3	7515	6887	✓	x	x
796	R1	8891	✓	x	x
796	R3	8881	✓	x	x
796	9604	8837	✓	x	x
796	9147	8376	✓	x	x
796	8538	7761	✓	✓	✓
796	7795	7018	✓	✓	x
796	7515	6737	✓	✓	✓
836	R1	8851	✓	x	x
836	R3	8841	✓	x	x
836	9604	8797	✓	x	x
836	9147	8336	✓	x	x
836	8538	7721	✓	x	x
836	7795	6978	✓	x	x
836	7515	6697	✓	x	x
1127	R1	8560	✓	x	x
1127	R3	8550	✓	x	x
1127	9604	8506	✓	x	x
1127	9147	8045	✓	x	x
1127	8538	7430	✓	x	x
1127	7795	6687	✓	x	x
1127	7515	6406	✓	x	x
1546	R1	8141	✓	x	x
1546	R3	8131	✓	x	x
1546	9604	8087	✓	x	x
1546	9147	7626	✓	x	x
1546	8538	7011	✓	✓	✓
1546	7795	6268	✓	✓	✓
1546	7515	5987	✓	✓	✓
2078	R1	7609	x	x	x
2078	R3	7599	x	x	x
2078	9604	7555	x	x	x
2078	9147	7094	x	x	x
2078	8538	6479	x	x	x
2078	7795	5736	x	x	x
2078	7515	5455	x	x	x
711	8538	7867	✓	x	x
711	8862	8200	✓	x	x
711	8882	8214	✓	x	x
711	8966	8300	✓	x	x
711	9039	8372	✓	x	x
711	9081	8416	✓	x	x
756	8538	7822	✓	✓	✓
756	8862	8155	✓	x	x
756	8882	8169	✓	x	x
756	8966	8255	✓	✓	✓
756	9039	8327	✓	x	x
756	9081	8371	✓	x	x
796	8538	7761	✓	✓	✓
796	8862	8094	✓	x	x
796	8882	8108	✓	x	x
796	8966	8194	✓	x	x
796	9039	8266	✓	x	x
796	9081	8310	✓	x	x
**Second-round primer sets**					
**Forward**	**Reverse**	**Amplicon size (bp)**	**Amplification**	**Single Band**	**Specificity**
756	8966	8255	✓	x	x
756	8538	7822	✓	x	x
796	8966	8194	✓	x	x
796	8538	7761	✓	✓	✓

Primer sets consisting of HIV-1 published and custom-designed primers were scored based on length of amplicon (bp) and amplification, sensitivity, and non-specific binding of NL4-3.Luc.E- and NL4-3.AD8 plasmids as positive controls, uninfected CD4^+^ T cells, and a non-template control. Coordinates of primers are based on HXB2 reference sequence. ✓: yes, x: no.

**Table 3:** First- and second-round primer sets for short (5′-end) HIV-1 amplification.

**First Round Primer Sets**					
**Forward**	**Reverse**	**Amplicon Size (bp)**	**Amplification**	**Single Band**	**Specificity**
711	6342	5672	✓	x	x
711	6214	5544	✓	x	x
711	6207	5542	✓	x	x
711	5857	5194	✓	x	x
711	5833	5172	✓	x	x
711	5562	4892	✓	x	x
711	5195	4528	✓	x	x
711	4956	4287	✓	x	x
711	4176	3506	✓	x	x
711	3501	2835	✓	x	x
711	3299	2629	✓	x	x
711	3246	2577	✓	x	x
711	2872	2202	✓	x	x
711	2691	2027	✓	x	x
711	2603	1934	✓	x	x
711	2302	1636	✓	x	x
756	6342	5627	✓	x	x
756	6214	5499	✓	x	x
756	6207	5497	✓	x	x
756	5857	5149	✓	x	x
756	5833	5127	✓	x	x
756	5562	4847	✓	x	x
756	5195	4483	✓	x	x
756	4956	4242	✓	x	x
756	4176	3461	✓	✓	✓
756	3501	2790	✓	✓	✓
756	3299	2584	✓	x	x
756	3246	2532	✓	✓	✓
756	2872	2157	✓	x	x
756	2691	1982	✓	✓	✓
756	2603	1889	✓	✓	✓
756	2302	1591	✓	✓	✓
**Second Round Primer Sets**					
**Forward**	**Reverse**	**Amplicon size (bp)**	**Amplification**	**Single Band**	**Specificity**
756	2302	1591	✓	x	x
796	2302	1530	✓	x	x
796	2138	1362	✓	x	x
796	1960	1186	✓	✓	✓

Primer sets consisting of HIV-1 published and custom-designed primers were scored based on length of amplicon (bp) and amplification, sensitivity, and non-specific binding of NL4-3.Luc.E- and NL4-3.AD8 plasmids as positive controls, uninfected CD4^+^ T cells, and a non-template control. Coordinates of primers are based on HXB2 reference sequence. ✓: yes, x: no.

**Table 4:** Patient characteristics.

	Patient 1	Patient 2
Sex	Male	Male
Age	71	70
Viral load (RNA/mL)	<40	<40
Suppressed for (years)	18	16
CD4 count (cells/μL)	1017	588

**Table 5:** Immortalization and HIV-positive wells recovery efficiency.

	Patient 1	Patient 2
Total plated cells	34,560	161,280
Total plated wells	1728	8064
Immortalized wells	1345	2768
HIV-positive wells	19	35

**Table 6:** Primers used for HIV-1 sequencing.

Primer Location	Sequence (5′-3′)
+796	GCGAGAGCGTCAGTATTAAGC
+836	GGGAAAAAATTCGGTTAAGGCC
+1127	AAAAGGCACAGCAAGCAGCAGCT
−1631	TTTGGTCCTTGTCTTATGTCCAGAATGC
+1546	AATCCACCTATCCCAGTAGGAGAAAT
−1960	CTTTGCCACAATTGAAACACTT
+2078	AGGCTAATTTTTTAGGGA
+2165	CAGAAGAGAGCTTCAGGTTTGGG
−2138	TGTTGGCTCTGGTCTGCTCT
−2302	CTAATAGAGCTTCCTTTAGTTGCC
−2603	GGCCATTGTTTAACTTTTGGG
+2603	CCCAAAAGTTAAACAATGGCC
−2691	TATGGATTTTCAGGCCCAATTTTTGA
−2872	TGCATCACCCACATCCAGTA
−3246	CCATTTATCAGGATGGAGTTC
−3299	TGTCATTGACAGTCCAGCTG
+3194	CACACCAGACAAAAAACATCAG
+3379	RGCAATTATGTAAACTCCTTAGGGGA
−3501	TAAGTCTTTTGATGGGTCATAATA
+3869	CTATGTAGATGGGGCAGCTA
−4176	TCTACTTGTTCATTTCCTCC
+4024	AAGTAAACATAGTAACAGACTCAC
+4162	CACACAAAGGAATTGGAGGAAATG
+4745	TAAGACAGCAGTACAAATGGCAG
−4956	TACTGCCCCTTCACCTTTCCA
−5195	TAGTGGGATGTGTACTTCTGAAC
+5550	AGAGGATAGATGGAACAAGCCCCAG
−5562	TGGTCTTCTGGGGCTTGTTC
−5833	AGGGCTCTAGTCTAGGATCTACTGGCTCCA
−5857	GGCTGACTTCCTGGATGCTTCCAGGGC
+5861	TGGAAGCATCCAGGAAGTCAGCCT
−6207	CTCTCATTGCCACTGTCTTCTGCTC
+6210	CAGAAGACAGTGGCAATGA
−6214	CACTCTCATTGCCACTGTCT
−6342	TTCCACACAGGTACCCCATA
+6445	GTGTACCCACAGACCCCAACCCACAAG
−6952	TTCCATGTGTACATTGTACTGTG
+6952	GCACAGTACAATGTACACATGGAA
−7515	GGCATACATTGCTTTTCC
+7515	GGAAAAGCAATGTATGCC
−7795	ATAGTGCTTCCTGCTGCTC
+7626	TTCAGACCTGGAGGAGGAGATATG
+7795	GAGCAGCAGGAAGCACTAT
+7850	ACAATTATTGTCTGGTATAGTGCAACAGCA
−8538	CAATCAAGAGTAAGTCTCT
+8277	TTCATAATGATAGTAGGAG
−9146	CTGCCAATCAGGGAAGTAGCCTTGTGT
−9604	GCACTCAAGGCAAGCTTTATTGAGGCTTA

Coordinates based on HXB2 reference sequence; + and − symbols indicate primer orientation.

## Data Availability

The original contributions presented in this study are included in the article/Supplementary Materials. Further inquiries can be directed to the corresponding author.

## Supplementary Materials

The following supporting information can be downloaded at https://www.mdpi.com/article/10.3390/ijms27021086/s1.
