# Supplementary figures and images for: Immortalization and Targeted Enrichment of HIV-Infected CD4+ T-Cells from Patients Under Antiretroviral Therapy
Source: Int J Mol Sci. 2026 Jan 22;27(2):1086. doi: 10.3390/ijms27021086 (PMC12841691; doi:10.3390/ijms27021086)

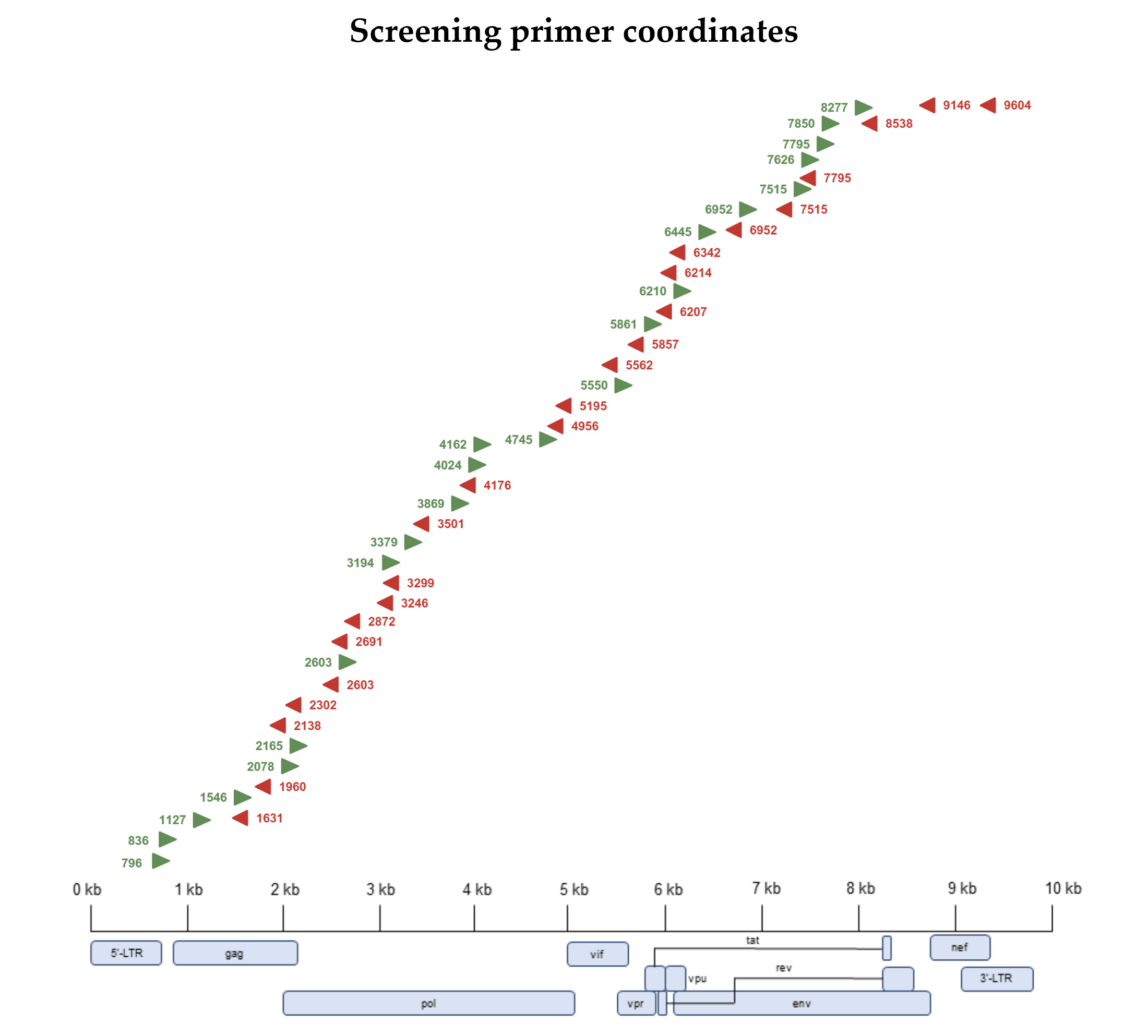

Supplement: Supplementary file 1 [file ijms-27-01086-s001.zip › Supplementary Figure S1.tif]

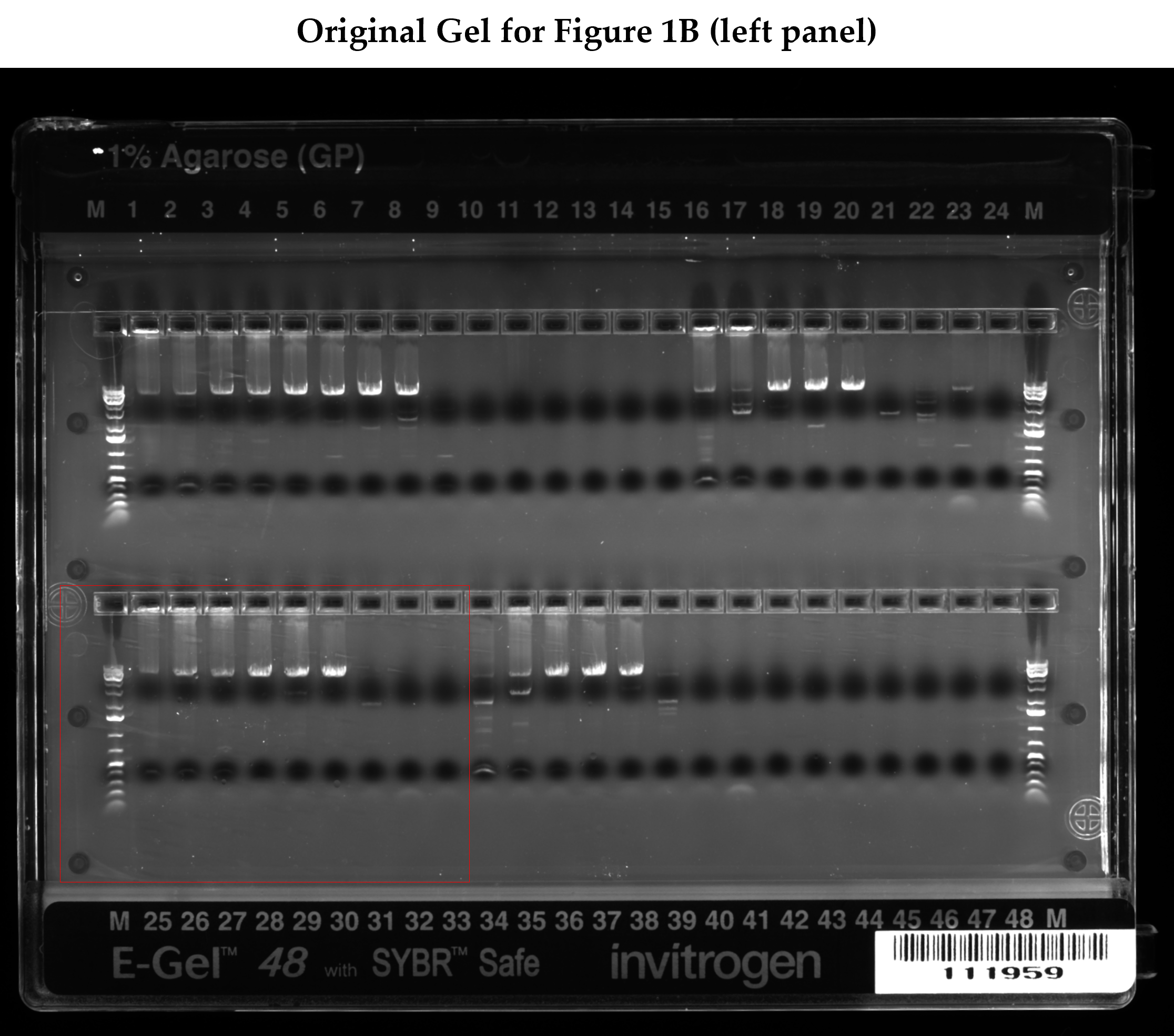

Supplement: Supplementary file 1 [file ijms-27-01086-s001.zip › Supplementary Figure S2.tif]

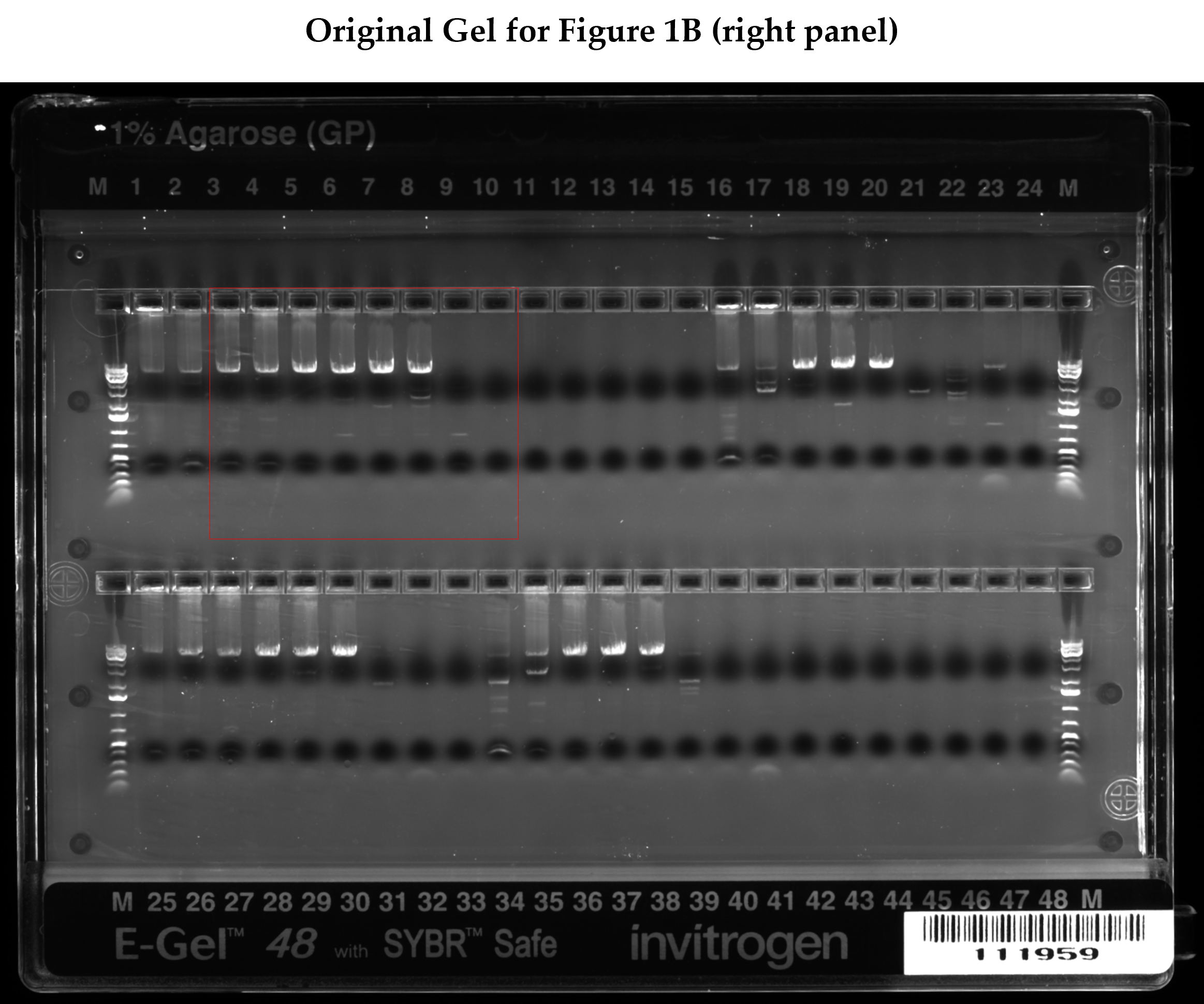

Supplement: Supplementary file 1 [file ijms-27-01086-s001.zip › Supplementary Figure S3.tif]

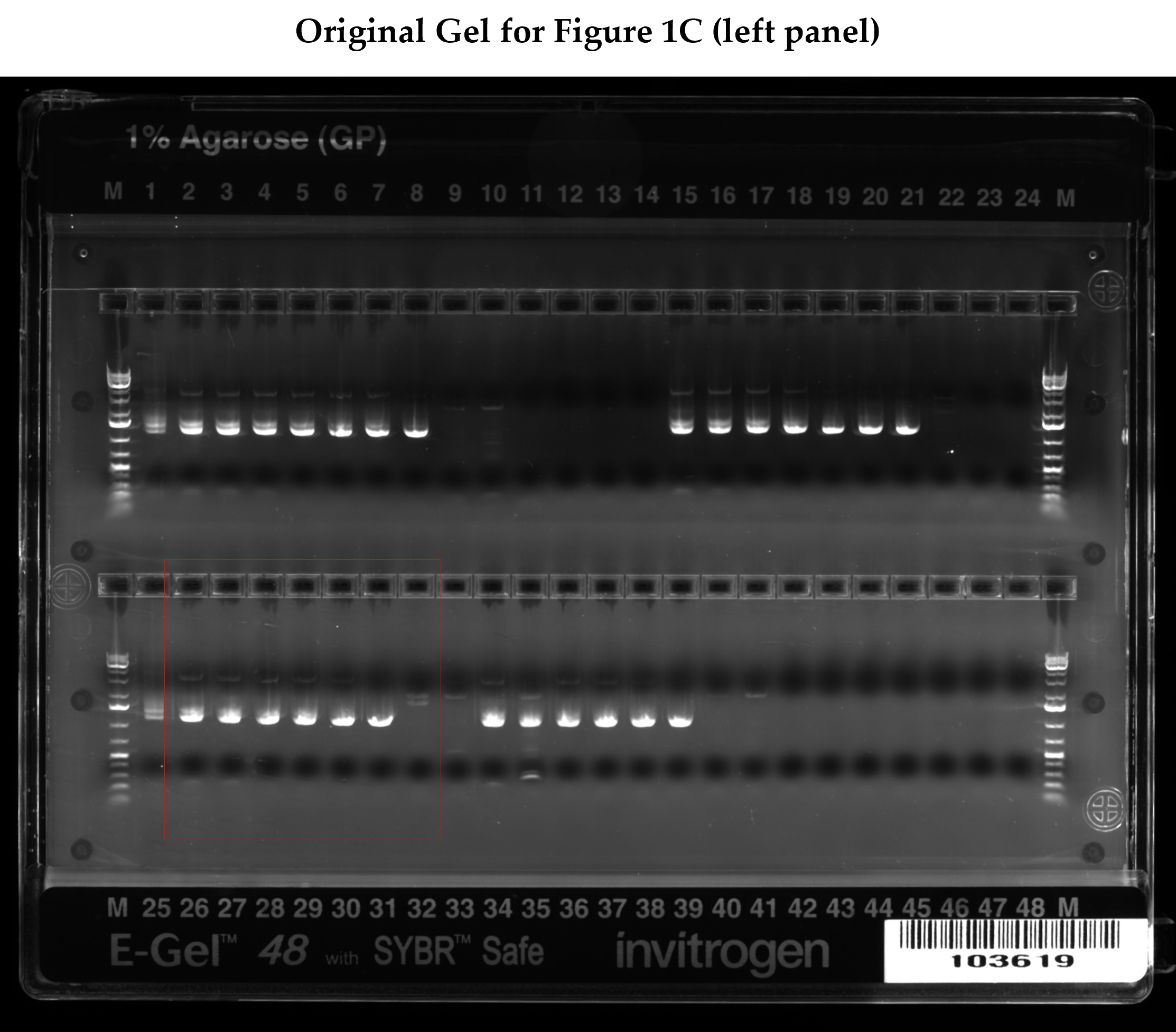

Supplement: Supplementary file 1 [file ijms-27-01086-s001.zip › Supplementary Figure S4.tif]

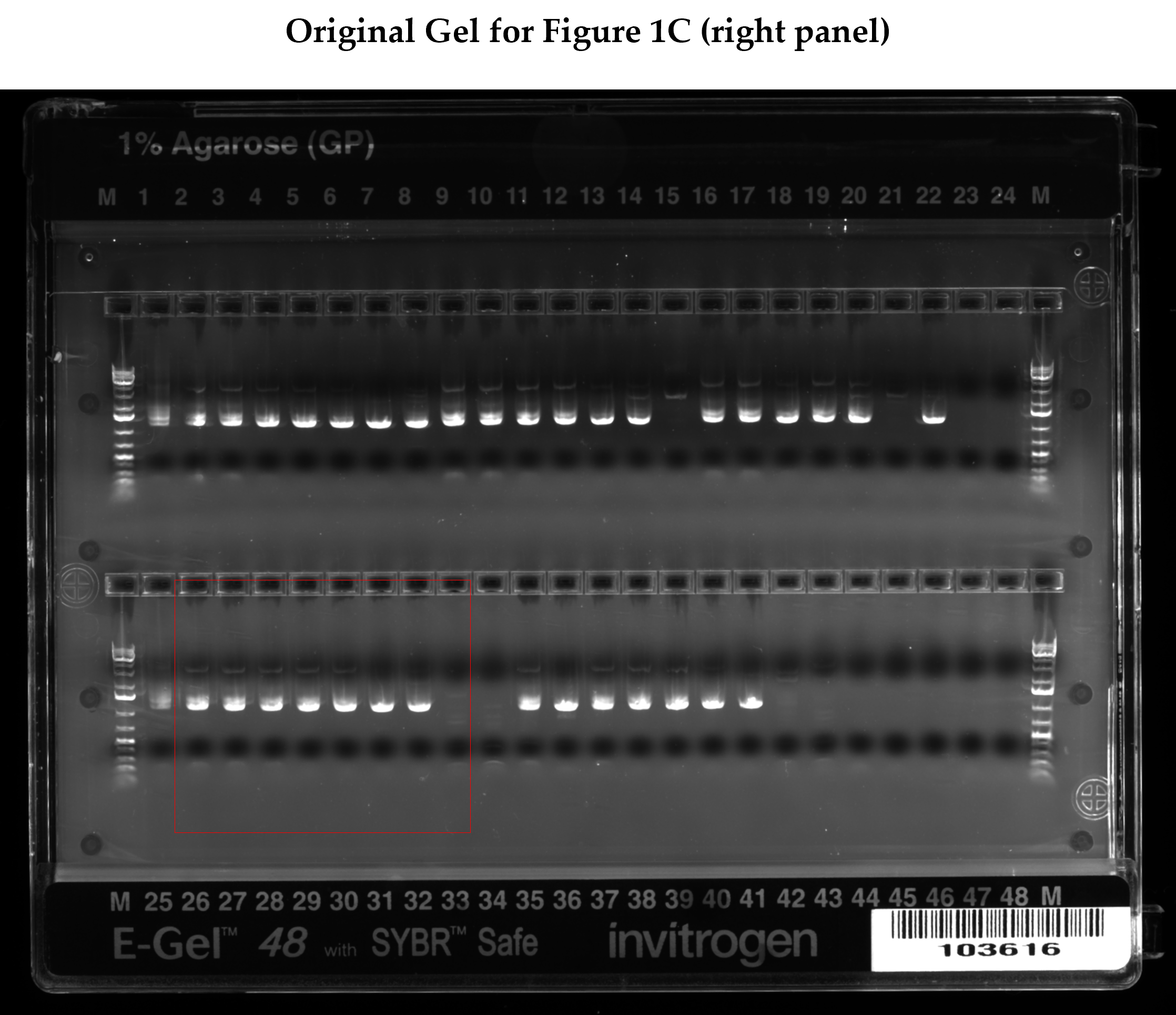

Supplement: Supplementary file 1 [file ijms-27-01086-s001.zip › Supplementary Figure S5.tif]

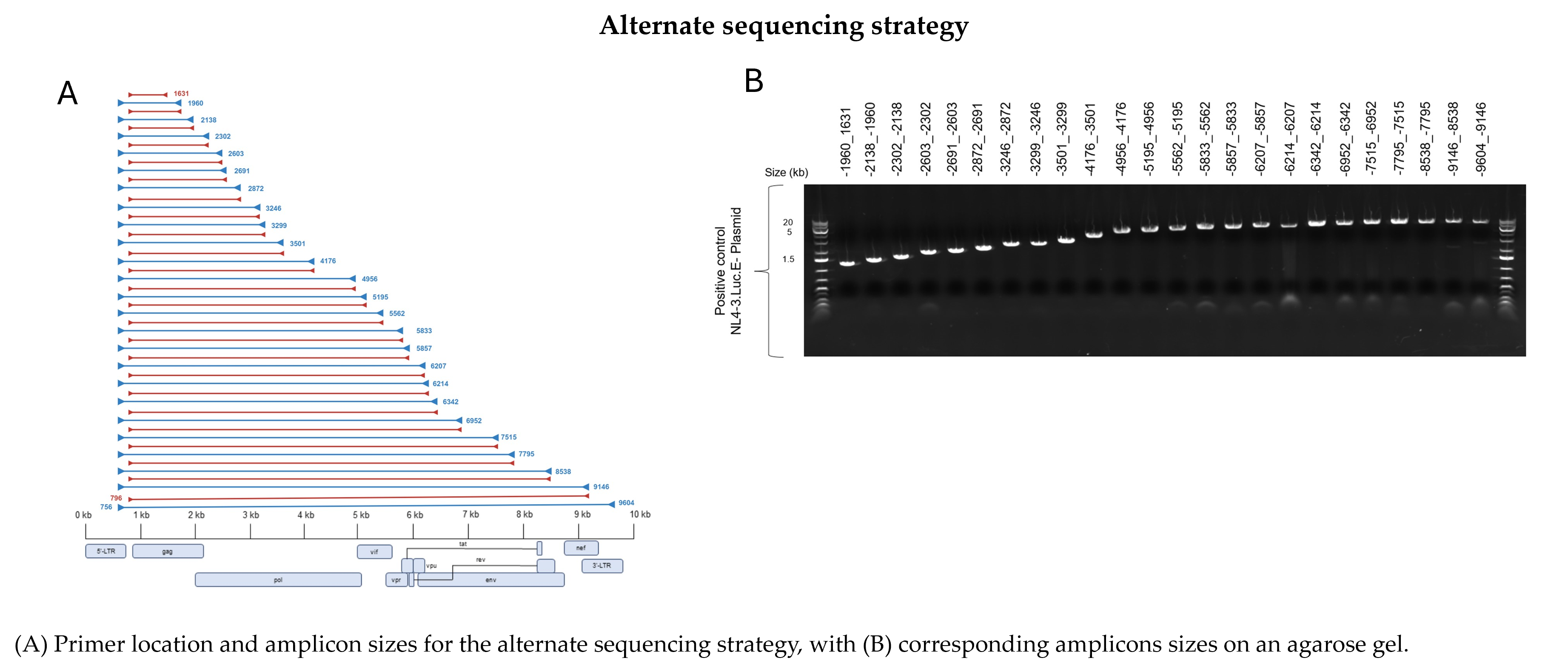

Supplement: Supplementary file 1 [file ijms-27-01086-s001.zip › Supplementary Figure S6.tif]

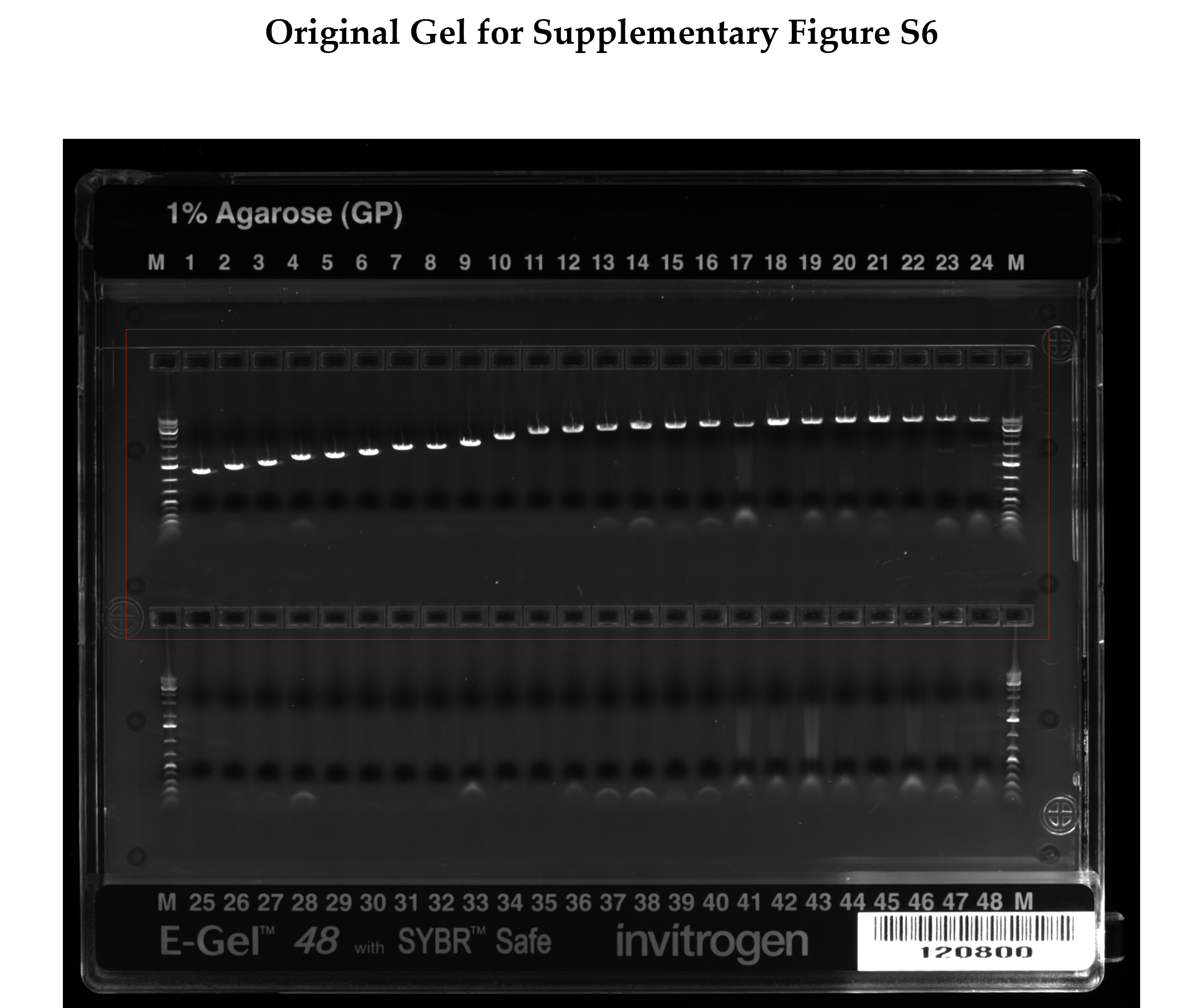

Supplement: Supplementary file 1 [file ijms-27-01086-s001.zip › Supplementary Figure S7.tif]

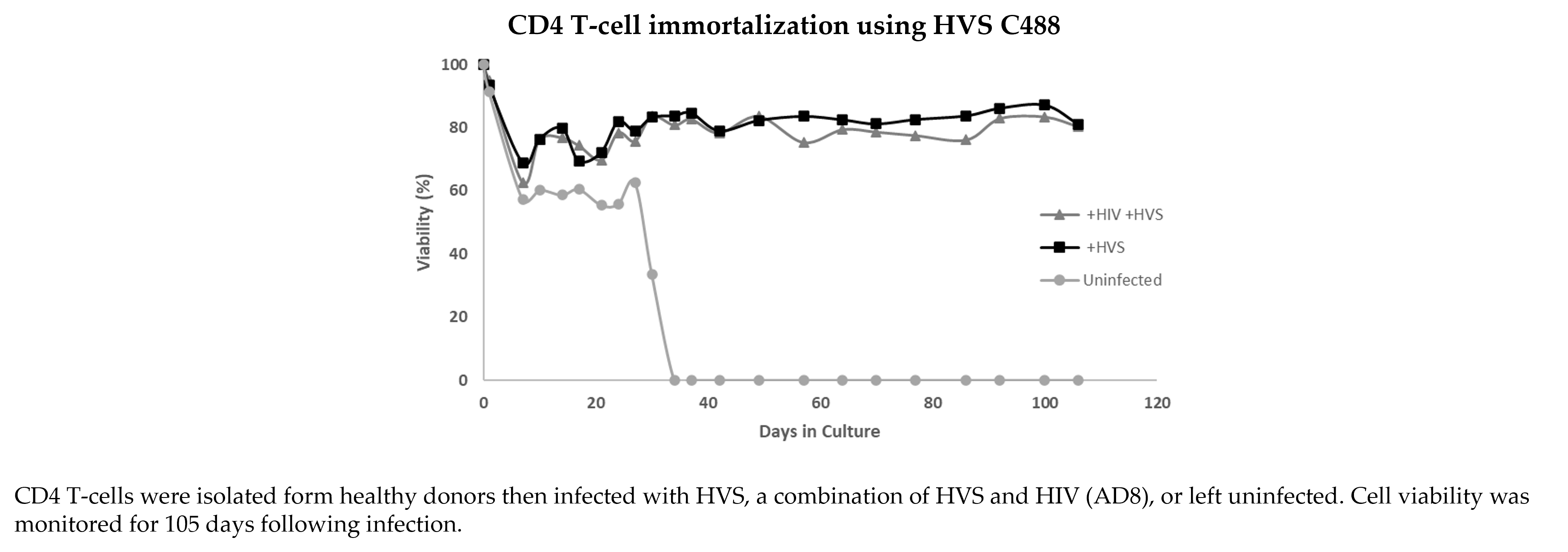

Supplement: Supplementary file 1 [file ijms-27-01086-s001.zip › Supplementary Figure S8.tif]
